# Effect of Fiber Sizing Levels on the Mechanical Properties of Carbon Fiber-Reinforced Thermoset Composites

**DOI:** 10.3390/polym15244678

**Published:** 2023-12-11

**Authors:** Albraa A. Jaber, Sara A. Abbas, Abdiaziz A. Farah, Karina K. Kopeć, Yahya M. Alsalik, Mohammed A. Tayeb, Nikhil Verghese

**Affiliations:** SABIC Technology Center (STC-K), King Abdullah University of Science and Technology (KAUST), P.O. Box 4545-4700, Thuwal 23955-6900, Saudi Arabia

**Keywords:** carbon fiber, thermoset, sizing, single fiber tensile testing, single fiber pullout testing, sizing levels, X-ray photoelectron spectroscopy (XPS)

## Abstract

Fiber sizing is one of the most important components in manufacturing composites by affecting mechanical properties, including strength and stiffness. The sizing of manmade fibers offers many advantages, such as improving fiber/matrix adhesion and bonding properties, protecting fiber surfaces from damage during the processing and weaving stages, and enhancing the surface wettability of polymer matrices. In this work, the influence of fiber sizing levels on carbon fibers’ (CFs) mechanical properties is reported at room temperature using single fiber tensile testing (Favimat+), single fiber pullout testing (SFPO), and interfacial elemental analysis by X-ray photoelectron spectroscopy (XPS). Standard modulus CFs (7 ± 0.2 μm in diameter) were sized using two commercially available Michelman sizing formulations. The average solid content for each sizing formulation was 26.3 ± 0.2% and 34.1 ± 0.2%, respectively. HEXION RIMR 135 with curing agent RIMH 137 was used as a model thermoset epoxy matrix during SFPO measurements. A predictive engineering fiber sizing methodology was also developed. Sizing amounts of 0.5, 1, and 2 wt.% on the fiber surface were achieved for both sizing formulations. For each fiber size level, 50 single-fiber tensile testing experiments and 20 single-fiber pull-out tests were conducted. The ultimate tensile strength (*σ_ult_*) of the carbon fibers and the interfacial shear strength (*τ_app_*) of the single fiber composite were analyzed. The sizing levels’ effect on interfacial shear stress and the O/C (Oxygen/Carbon) surface composition ratio was investigated. Based on our experimental findings, an increase of 6% in fiber performance was recorded for ultimate tensile and interfacial shear strengths. As a result, generalized fiber sizing and characterization methods were established. These developed methods can be used to characterize the strength and interfacial shear strength of manmade fibers with different sizing formulations and solid contents irrespective of the matrix, i.e., thermoset or thermoplastic.

## 1. Introduction

In the last few decades, the research and manufacturing of manmade fibers such as carbon, glass, and aramid fibers have gained much interest for their versatility [1,2,3,4,5,6]. Notably, carbon fiber-reinforced polymers (CFRPs) represent the highest-performance polymer-matrix composites in aircraft components, fuel-efficient automobiles, high-performing machinery, improved construction materials, sustainable sources of energy components, and new materials for smart infrastructure [7,8,9,10,11,12]. Furthermore, carbon fibers have been made from many different precursors, such as polyacrylonitrile (PAN), pitch, rayon, polyethylene, and lignin. PAN and pitch are the most favorable CF precursors for industrial applications, with PAN accounting for approximately 90% of all production [6,13,14,15,16,17,18,19]. Additionally, CFRPs preserve their high tensile moduli and strengths even in harsh environments with elevated temperatures, offering excellent electrical and thermal conductivity and having a relatively low coefficient of thermal expansion [1]. However, advancing carbon fiber properties has proven challenging under carbon fiber production complexity. The process of designing and synthesizing polymer precursors to convert these polymers into carbon fiber with desired properties is influenced by dozens of parameters. Ultimate carbon fiber performance also relies on the appropriate design of the precursor chemistry and structure. The creation of carbon fiber with appropriate qualities begins with developing and synthesizing polymer precursors, which is a process controlled by numerous factors. Additionally, it is well acknowledged that the chemistry and structure of the precursors must be properly designed for the carbon fiber to display the intended specifications.

The interfacial adhesion between the fiber and matrix is paramount importance to designing and producing the intended fiber application for high-performing functional composite. Several techniques exist to accomplish this, including surface treatment and sizing the reinforcing fibers while the product is being manufactured [20,21,22,23]. The process phase when a thin film of organic polymer is placed onto the fiber surface is known as the “sizing” of carbon fibers. Manmade fibers can be surface treated and sized for various benefits, including increased surface wettability of the polymer matrices, enhanced fiber/matrix adhesion and bonding qualities, and protection from damage during processing and weaving.

Fiber-matrix interface studies have gained much research focus during the last decade, particularly with the increased use of thermoplastic resins as matrices [24,25,26,27]. The interface is the boundary area that leads to stress transfer from one CF filament to another through the matrix [19,24,25,26,27]. Interfacial adhesion can follow various mechanisms that include chemical bonding and mechanical bonding. If the interface layer area between the fiber and the matrix is weak, poor mechanical properties will be observed due to the lack of adhesion [24,25,26,27]. On the contrary, if the matrix and the CF adhesion are strong, the final composite will be brittle. Consequently, interfacial adhesion is an optimization effort and not one of maximization. Surface engineering is therefore highly required to achieve the optimum adhesion level. Researchers have looked at how carbon fiber surface treatment and size affect the interfacial characteristics of various polymeric matrices [20,21,22,23,28]. Kamps et al. [21] investigated the impact of electrolytic surface treatment parameters, such as current, voltage, and conductivity, on the adhesion characteristics of carbon fiber-reinforced polycarbonate composites. Due to the considerable increase in polarity and the presence of hydroxyl, carboxyl, and nitrile groups on the fiber surface, Kamps et al.’s methods effectively demonstrated a 12% increase in apparent interfacial shear strength [21]. Other investigations into the impact of size on carbon fiber adhesion to the epoxy matrix were also conducted by Zhang [19] and Drzal et al. [29]. Both teams concluded that the sizing layer had increased shear strength by 14%. The impact of carbon fiber oxidization parameters and sizing deposition levels on the fiber-matrix interfacial shear strength of unsized and sized fibers has also been studied by Stojcevski et al. [30]. They determined the interfacial shear strength (IFSS) of single monofilaments using a two-epoxy resin system as a matrix. The IFSS increased by 56%, according to Stojcevski et al. IFSS is correlated with increasing current, but proper sizing is required for best performance.

In this study, our efforts focused on assessing the fiber–matrix interaction at various sizing degrees (i.e., 0.5, 1, and 2 wt.%). In this study, we aimed to close the gap, investigate the sizing design space, and shed light on how different size levels affect the mechanical properties of fiber. To accomplish this, a specialized fiber-sizing machine was used to apply two thermoset commercially available polymer sizing dispersions to the carbon fiber roving. Each dispersion was created with the necessary solid content (three dispersions each). We investigated how the size level affected interfacial elemental analysis, fiber–epoxy adhesion, and single-fiber tensile strength.

## 2. Materials

### 2.1. Carbon Fiber

**Figure 1:**
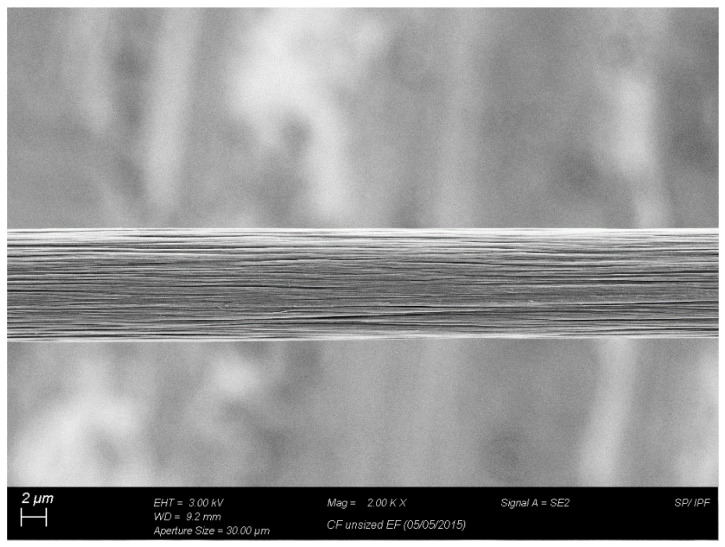
Scanning electron microscopy (SEM) image of the CN carbon fiber.

**Figure 2:**
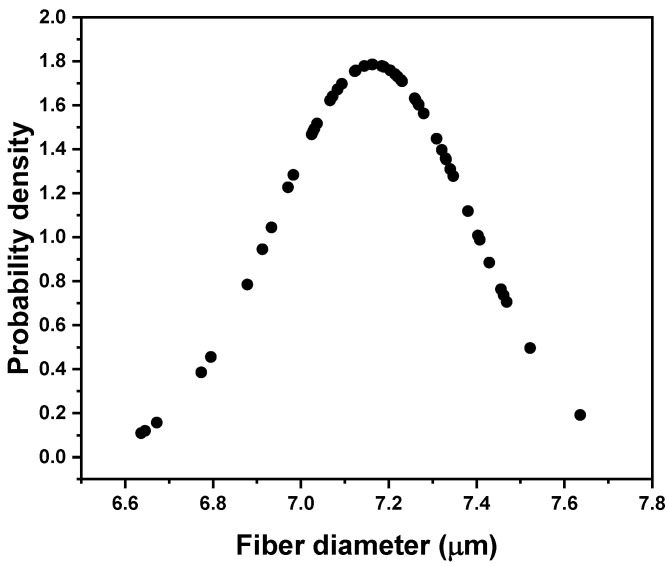
Measured size distribution of the carbon fibers, 7 ± 0.2 μm.

For the fiber sizing experiments, and commodity-related applications [21], polyacrylonitrile (PAN) unsized, standard modulus, and standard strength carbon fiber supplied by Carbon Nexus (Waurn Ponds, Australia) was used as a reinforcement fiber. Figure 1 shows a scanning electron microscopy (SEM) image under 2000× of the carbon fiber. The average fiber diameter was measured to be 7 ± 0.2 μm. Figure 2 shows the measured distribution of the CN fiber diameters used in this study.

### 2.2. Epoxy Resin

To conduct single-fiber pullout experiments, EPIKOTE^™^ resin MGS^™^ RIMR 135 and EPIKURE^™^ curing agents MGS RIMH 134–137 supplied by Westlake Epoxy (Henderson, NV, USA) were used as a model thermoset system. The epoxy resin to hardener mixing ratio was (100:30 ± 2 by weight), and the resin mixture was cured at a temperature of 70 °C for 8 h. Table 1 highlights the mechanical properties of the cured thermoset.

### 2.3. Sizing Formulations

**Figure 3:**
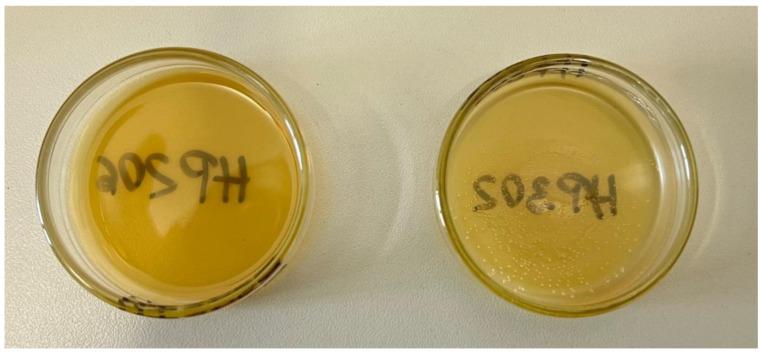
Sizing formed films after evaporating the dispersion medium. Left sample: Hydrosize^®^ HP2-06; right sample: Hydrosize^®^ HP3-02.

Two thermoset-compatible sizing formulations were identified, namely Hydrosize^®^ HP2-06 and Hydrosize^®^ HP3-02, supplied by Michelman^®^, Inc. (Cincinnati, OH, USA). The average manufacturer reported solid content for each sizing formulation as 26.3 ± 0.2% and 34.1 ± 0.2%, respectively. Figure 3 shows the films formed from the sizing formulation after the dispersion medium has evaporated.

## 3. Methods

**Figure 4:**
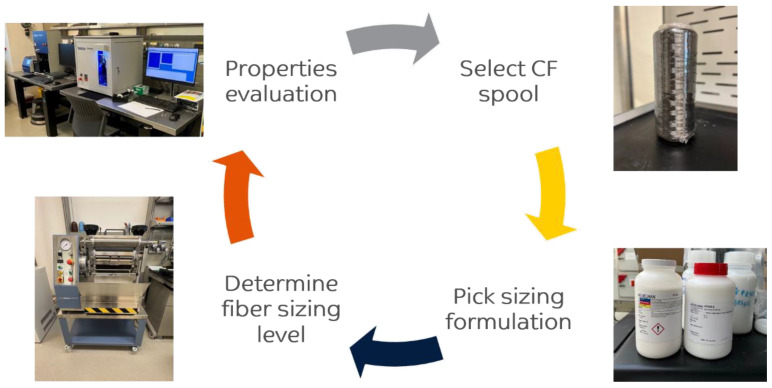
Overall experimental methodology.

Figure 4 shows our overall experimental methodology. The process starts with selecting a carbon fiber spool and identifying the desired sizing formulation and sizing levels. Afterward, the reinforcement fibers are sized and characterized using different techniques, such as single-fiber tensile testing, single-fiber pullout testing, and elemental analysis mapping using X-ray photoelectron spectroscopy (XPS).

### 3.1. Fiber Sizing Procedure

The fiber sizing process is the third step after selecting the reinforcement fiber spool and choosing the sizing formulation, as shown in Figure 4. To achieve 0.5, 1, and 2 wt.% of fiber sizing levels, the procedure starts with diluting the sizing formulation with deionized (DI) water following Equation (1). Equation (1) was developed as a function of the targeted sizing solid content level in [%], diluted sizing solution mass (sizing + DI water) in [g], and manufacturer sizing formulation solid content in [%]. The diluted sizing solution mass was fixed to 200 g, while for space mapping, the targeted sizing solid content on the fiber surface varied from 2 to 4 wt.%. Table 2 shows a breakdown of the sizing solution preparation using Equation (1). After preparing the diluted sizing solution, we used a horizontal and vertical padder type (HVF) supplied by Mathis (GER) to size the fiber tows. During the fiber sizing process, the pressure of the rollers was set to 1 bar, while the sizing speed was fixed at 0.80 m/min. These parameters align with our previously published effort in [21,22]. Lastly, the sized fiber tows were dried using a Heraeus UT6760 forced convection oven supplied by Thermo (Waltham, MA, USA) at 160 °C for 4 h.

(1) $$Targeted\;lot\;mass\;\left[g\right]=\left(\frac{Targeted\;sizing\;solid\;content\;\left[\%\right]\times diluted\;sizing\;solution\;mass\;\;\left[g\right]}{manufacturer\;sizing\;formulation\;solid\;content\;\left[\%\right]}\right)$$

### 3.2. Determination of Fiber Sizing Content

The amount of fiber sizing was determined following the DIN ISO 1887 standard [31]: sizing content determination by loss on ignition (LOI) at 650 °C. LOI experiments were conducted using the Phoenix Airwave microwave muffle furnace in air supplied by CEM Corporation (Stallings, NC, USA). Equation (2) was used to calculate the fiber sizing content in (wt.%) as a function of the mass of the pan in [g], the mass of the sample in [g], and the mass of the sample after ashing in [g].

(2) $$Fiber\;sizing\;content=\left[\frac{\left(m_{Pan}+m_{Sample}\right)-m_{sample\;after\;ashing}}{\left(m_{Pan}+m_{Sample}\right)-m_{pan}}\right]\times 100$$

### 3.3. Single Fiber Tensile Testing

Single-fiber tensile testing experiments for both unsized and sized carbon fibers were conducted using a Favimat+ single-fiber tester supplied by Textechno H. Stein GmbH & Co. KG (Mönchengladbach, Germany). The tensile load extension curves were collected at a cross-head rate of 15 mm/min using a gauge length of 50 mm and a pretension of 2 cN/tex. The specific tensile strength (ultimate specific stress or tenacity) and modulus were determined by normalizing the load data and dividing by the linear density to provide specific stress-strain curves. The breaking stress of the fiber was obtained by dividing the maximum recorded force by the fiber’s area.

### 3.4. Fiber–Matrix Adhesion: Single Fiber Pull-Out Testing

**Figure 5:**
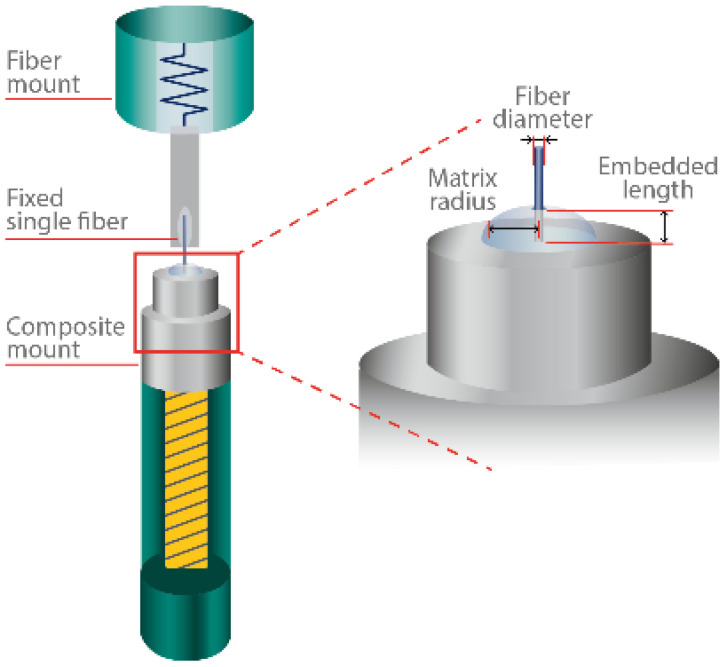
Single fiber pull-out test setup.

The interfacial adhesion strength between the fiber and matrix of unsized and sized fiber was evaluated using a custom-made single-fiber pull-out (SFPO) instrument and purpose-built embedding equipment constructed by IPF Dresden, (Germany) [32,33], as shown in Figure 5. A pre-selected embedding sample length of ($l_{e}=100\;\mu m$) was prepared and embedded accurately and perpendicularly to the surface of the epoxy matrix. We set an embedding temperature of 85 °C under a controlled atmosphere and temperature for the epoxy formulation. After embedding, the epoxy formulation was cured at 85 °C for about 10 s before cooling down to ambient temperature, after which the pull-out test was conducted with a loading rate of 10 nm/s. The force-displacement curves and the maximum force (*F_max_*) required for pulling the fiber out of the matrix were measured. After testing, the fiber diameter (*d_f_*) was measured using an optical microscope; *l_e_* was determined using the force-displacement curve and cross-checked using a scanning electron microscope (SEM). The adhesion bond strength between the fiber and the matrix was characterized by the apparent interfacial shear strength values presented in Equation (3) [21,32,33,34].

(3) $$\tau_{app}=\left[\frac{F_{max}}{\left(\pi \times d_{f}\times l_{e}\right)}\right]$$

### 3.5. X-ray Photoelectron Spectroscopy

We performed X-ray photoelectron spectroscopy on the prepared materials for core-level analysis. The XPS of unsized and sized fibers was conducted using the Thermo Scientific ESCALAB 250 Xi. The machine was equipped with a mono-chromated AlK_α_ X-ray source. The base pressure of the chamber was typically in the mid 10^−10^ mbar. Charge neutralization was used for all samples (compensating for shifts of ~1 eV). The spectra were calibrated with respect to C1s peak maxima at 284.8 eV. The C1s, O1s, and N1s binding energy regions were scanned for all carbon fibers. Typical acquisition conditions were as follows: First, the pass energy and scan rate were set to 20 eV and 0.1 eV per 200 ms, respectively. The fiber samples were cut into squares with a dimension of 0.5 × 0.5 cm^2^, which were then loaded into the chamber for analysis. A typical spatial area analyzed was 0.9 × 0.9 mm^2^. Data acquisition and analysis were performed using AVANTAGE software V5.967.

## 4. Results and Discussions

### 4.1. Achieving the Targeted Sizing Level

The effect of carbon fiber sizing level on carbon fiber/epoxy composites is an important factor in its manufacturing. A higher sizing level can increase interfacial bonding between the carbon fibers and epoxy resin, improving mechanical properties such as strength and stiffness. However, excessive sizing can also lead to increased stiffness of the fiber, which can reduce overall processability during downstream part prepreg, tape, and part manufacturing. Consequently, excessive sizing negatively affects composite performance. Therefore, carefully balancing the sizing level to achieve optimal mechanical performance and durability is essential.

**Figure 6:**
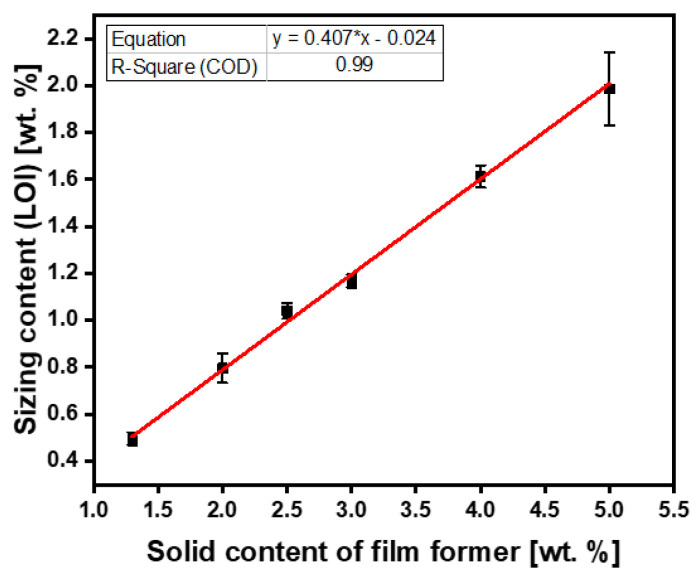
Hydrosize^®^ HP3-02 sizing experiments.

**Figure 7:**
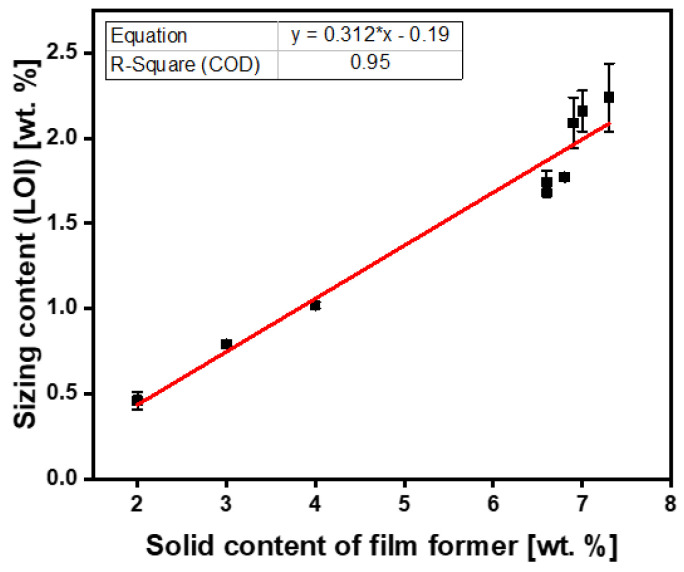
Hydrosize^®^ HP2-06 sizing experiments.

To quantify the effect of fiber sizing level on fibers’ mechanical properties, the desired fiber sizing levels must be achieved with a high degree of accuracy, namely (0.5, 1, and 2 wt.%). A series of three exploratory experiments with per-sizing formulations were conducted to determine the average fiber sizing level, as shown in Table 2. In Table 2, experiments A1–A3 and B1–B3 correspond to fiber sizing experiments using Hydrosize^®^ HP3-02 and Hydrosize^®^ HP2-06 commercial sizing formulations, respectively. The fiber-sizing experiments shown in Figure 6 and Figure 7 show the relationship between the sizing solid content of the film former and plot the sizing level from the LOI.

For Hydrosize^®^ HP3-02 formulation (Figure 6), the initial three sizing experiments resulted in LOI sizing levels of 0.80, 1.17, and 1.61 wt.% on the fiber’s surface. As a result, a first-order straight line fit with (R^2^ = 0.99) was obtained. As shown in Figure 6, these exploratory experiments yielded a predictive engineering approach to determine the desired fiber sizing level while eliminating the trial-by-error approach. Based on these findings, the desired predictive sizing level by LOI of the Hydrosize^®^ HP3-02 formulation can be calculated using Equation (4). Based on Equation (4), the updated solid content of the film former was calculated for experiments A4–A6, as shown in Table 3. Resizing the fibers with the recalculated predictive solid content of the film former (Equation (4)) at 1.29, 2.52, and 5.0 wt.% solid content resulted in 0.5, 1, and 2 wt.% of LOI sizing levels, as shown in Table 3 and Figure 6.

(4) $$Predictive\;sizing\;solid\;content_{HP3-02}\left[\%\right]=\left(0.407\times \left(desired\;fiber\;sizing\;level\right)\right)\left[\%\right]-2.40\times 10^{-2}$$

On the other hand, the three exploratory experiments for Hydrosize^®^ HP2-06 formulation (Table 2 and Figure 7) resulted in LOI sizing levels of 0.46, 0.81, and 1.02 wt.% on the fiber’s surface. Unlike the Hydrosize^®^ HP3-02 sizing exploratory experiments, two out of three Hydrosize^®^ HP2-06 exploratory experiments achieved the targeted sizing levels without the need for resizing, namely 0.5 and 1 wt.%. Following the same approach developed for Hydrosize^®^ HP3-02 formulation, a straight line fitting with (R^2^ = 0.95) was attained for the 2 wt.% sizing level, where the new desired predictive sizing solid content of the Hydrosize^®^ HP2-06 formulation could be calculated using Equation (5). Based on Equation (5), we calculated the updated solid content of the film former in experiment B8 for the 2.0 wt.% LOI, as shown in Table 3. Resizing the fibers with a 6.90% film former solid content resulted in a 2 wt.% of an LOI sizing level, as shown in Table 3 and Figure 7. The desired fiber sizing levels were achieved for both sizing formulations with a high degree of accuracy. Therefore, the effect of fiber sizing on the mechanical properties will be discussed in the next section. For the next section, samples A4–A6, and B1, B3, and B8 were selected to study the effect of fiber sizing levels on mechanical properties. It is worth mentioning that the developed fiber sizing methodology can be applied to manmade fibers (glass, aramid, etc.) of any shape (circular, bean, etc.) and sizing formulations with different sizing solid content.

(5) $$Predictive\;sizing\;solid\;content_{HP2-06\;}\;\left[\%\right]=\left(0.312\times \left(desired\;fiber\;sizing\;level\right)\right)\left[\%\right]-0.19$$

### 4.2. The Effect of Sizing Level on the Tensile Properties of the Fiber

To study the effect of sizing level on the fibers’ mechanical properties, a series of 50 single-fiber tensile testing experiments (n) were conducted for each fiber sizing level using Favimat+. The ultimate tensile strength ($\sigma_{ult}$) of the carbon fibers was analyzed using the two-parameter Weibull distribution according to Equation (6), where *P* is the cumulative probability of a filament’s failure at the applied stress (*σ*), shape parameter (*m*), and characteristic stress (at which 63.2% break) (*σ*_0_) [35]. The high values of the shape parameter (*m*) indicate a homogenous distribution of damages over the entire filament surface.

$$P\left(\sigma \right)=1-e^{-{\left(\frac{\sigma }{\sigma_{0}}\right)}^{m}}$$

**Figure 8:**
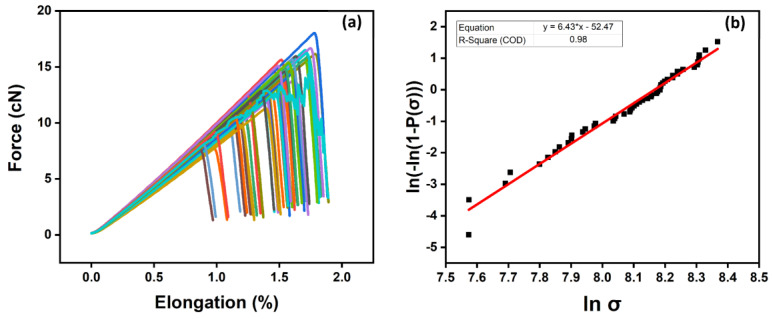
Unsized carbon fibers: (**a**) tensile testing measurement using FAVIMAT+; (**b**) two-parameter Weibull distribution analysis. The colors highlights a set of 50 broken fibers.

**Figure 9:**
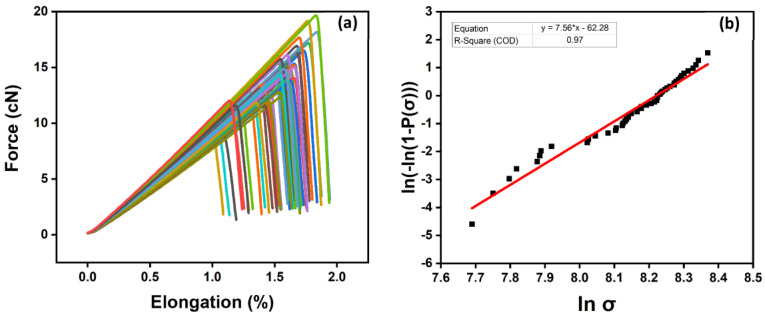
Representative sample (A5) of HP3-02-sized carbon fibers: (**a**) tensile testing measurement using FAVIMAT+; (**b**) two-parameter Weibull distribution analysis. The colors highlights a set of 50 broken fibers.

**Figure 10:**
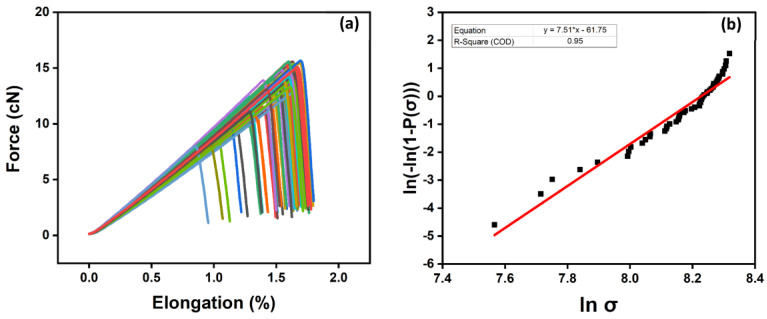
Representative sample (B1) of HP2-06-sized carbon fibers: (**a**) tensile testing measurement using FAVIMAT+; (**b**) two-parameter Weibull distribution analysis. The colors highlights a set of 50 broken fibers.

The effect of the carbon fiber sizing levels on the fiber’s tensile strength is shown in Table 4 and Figure 8a,b, Figure 9a,b and Figure 10a,b for an unsized representative sample (A5) of HP3-02-sized carbon fibers and a representative sample (B1) of HP2-06-sized carbon fibers, respectively. For the unsized fibers, Figure 8a shows the force vs. elongation tensile test measurements, while Figure 8b shows the two-parameter Weibull distribution analysis. As shown in Figure 8b and Table 4, the scale parameter ($\sigma_{0_{\;unsized}}$) of the unsized fiber is baselined at 3.52 GPa.

For HP3-02 sized fibers (Table 4), increasing the sizing content to 1 wt.% (see Figure 9, sample A5) resulted in a 6% increase in the scale parameter ($\sigma_{0_{\;1\;wt.\%}}$) to 3.72 GPa compared to unsized fibers. This observed increase in scale parameters can be attributed to covering fiber surface defects by the sizing formulation, which resulted in a more homogenous distribution of the load along the fiber axis. Table 4 also shows that a further increase in the fiber sizing level to 2 wt.% (sample A6) resulted in a 3% decrease in the scale parameter ($\sigma_{0_{\;2\;wt.\%}}$) to 3.43 GPa compared to the unsized fibers. This finding is primarily due to human-induced damage during the fiber separation process. The latter claim was also supported by the shape parameter (m), which was the lowest among its group at 5.34. Hence, although the fiber sizing level increased, handling and processing became more challenging.

Table 4 shows the effect of fiber sizing with Hydrosize^®^ HP2-06 formulation on fiber tensile strength. As the sizing level increased to 0.5 wt.% (see Figure 10, sample B1), the scale parameter ($\sigma_{0_{\;0.5\;wt.\%}}$) increased by more than 6% compared to unsized fibers. A further increase in the fiber sizing level decreased the scale parameter, as explained previously. Noticeably, HP2-06 sizing showed an optimized tensile strength performance at a lower solid content ($\sigma_{0}=3.74\;GPa$ at 0.5 wt.% sizing) compared to HP3-02 ($\sigma_{0}=3.72\;GPa$ at 1 wt.% sizing). This is a significant finding as it will add another economical dimension during the process of selecting an effective low-cost sizing agent in a real manufacturing environment. At the same time, increasing the sizing level to 2 wt.% negatively affects the post-processing stages (prepreg manufacturing) due to difficulties faced throughout the fiber spreading process. The effect of sizing on the surface functional groups of sized and unsized carbon fibers will be discussed in the next section.

### 4.3. The Effect of Sizing on Functional Groups

**Figure 11:**
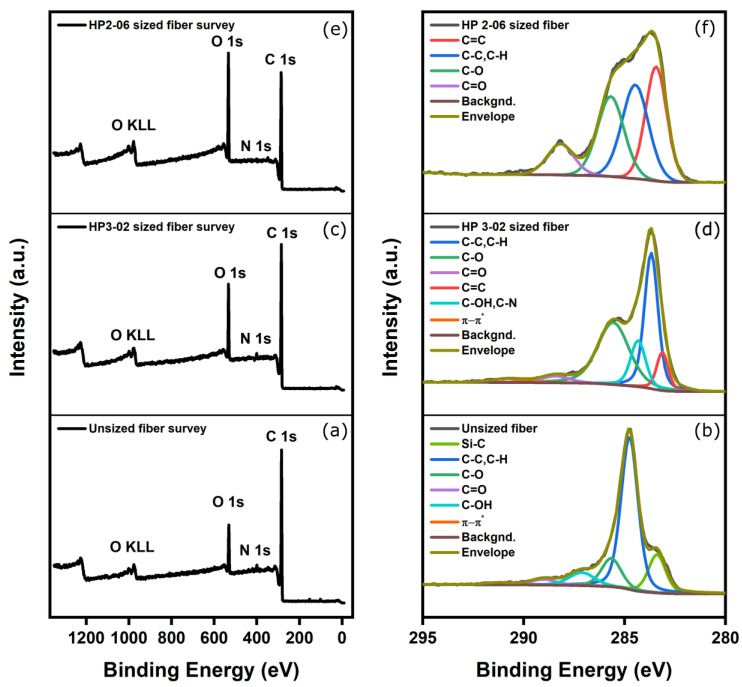
(**a**) XPS survey of unsized fibers; (**b**) high-resolution C1s spectrum of unsized fibers; (**c**) XPS survey of HP3-02 sized fibers; (**d**) high-resolution C1s spectrum of HP3-02 sized fibers; (**e**) XPS survey of HP2-06 sized fibers; and (**f**) high-resolution C1s spectrum of HP2-06 sized fibers.

The local surface chemical composition is critically important in discerning the type and number of functional groups on the surface region of differently-sized carbon fibers. X-ray photoelectron spectroscopy (XPS) was employed to probe the chemical composition and degree of surface modification of treated and untreated fibers. Figure 11a,c,e shows a representative example of the survey spectrum for the unsized and sized surfaces of samples A and B as well as the high-resolution spectra of C1s. The survey spectrum exhibits peaks at 99.7, 284.7, 400.3, and 532.0 eV, corresponding to the binding energies of Si 2p, C1s, N1s, and O1s, respectively. High-resolution XPS scans were also conducted to gain additional insight into the chemical composition of these samples. As shown in Figure 11a,c,e, the survey spectrum for the unsized and sized A and B samples had three major components detected across all the samples, namely, C, O, and N, while some samples had a minor presence and impurities of silicon and calcium. The presence of calcium can be attributed to possible contamination from sample handling, whereas the presence of silicon can be attributed to the silicon oil agent applied to the surface of the polyacrylonitrile precursor incorporated into the fiber’s structure, which was preserved by the carbonization process [28]. Table 5 summarizes the detailed XPS elemental compositions of unsized and sized carbon fiber surfaces.

The high-resolution C1s spectrum of unsized and sized fibers can be fitted with four peaks, which are related to individual contributions from different functional groups (see Figure 11b,d,f). The peak at 283.3 eV relates to a carbide group, possibly silicon carbide, a residual from the carbonization process [28]. Furthermore, the high-resolution scan for C1s spectrum also shows three deconvoluted component peaks with binding energies characteristic of the molecular units present on the treated and untreated surfaces of these fibers. This scan includes peaks at -C-C/C-H (284.8 eV), -C-O-C (285.7 eV), and -C-C=O (287.1 eV), respectively. In addition, the peaks at 289 and 291.3 eV are assigned to carboxyl functions or ester (-COO-) and satellite peaks ($\pi -\pi^{*}$), respectively. The O1s envelope was fitted with three main peaks centered at 530.8, 532.5, and 534.6 eV attributed to C=O, C-O-H, and C-O-C, respectively. Based on high-resolution scans and the elemental composition analysis presented in Table 5 for both sizing formulations, the [O]:[C] ratio across all the samples increased linearly after increasing the amount of sizing. As a result, greater fiber–matrix adhesion is expected. Notably, HP2-06 showed a higher [O]:[C] ratio at 0.33 compared with the HP3-02 formulation, which plateaued at 0.22.

### 4.4. The Effect of Sizing Levels on the Fiber’s Interfacial Properties

**Figure 12:**
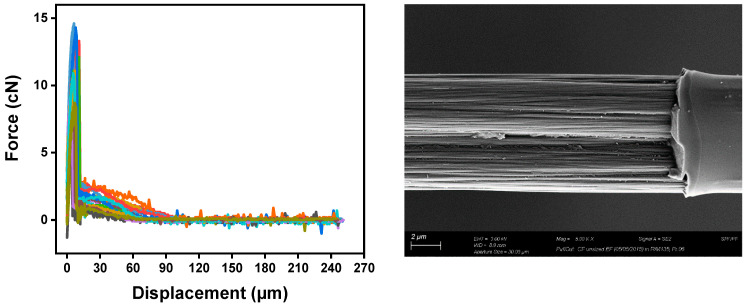
Force-displacement curves of unsized fibers (**left**) and SEM-image of a fiber after SFPO (**right**). The colors highlights a set of 20 broken fibers.

**Figure 13:**
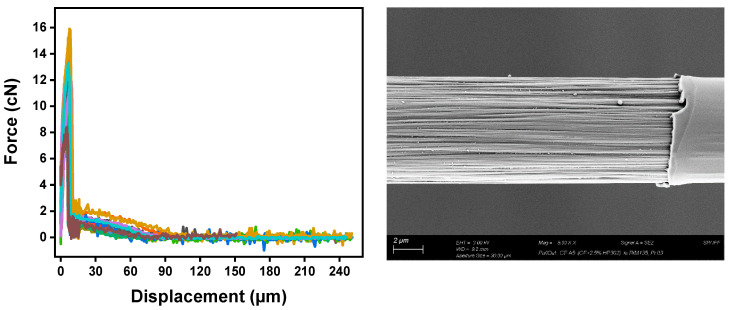
Representative sample (A5) of HP3-02 sized carbon fibers; (**left**) force-displacement curves of, and SEM observation of a fiber surface after testing showing a non-significant amount of residual epoxy on the surface (**right**). The colors highlights a set of 20 broken fibers.

**Figure 14:**
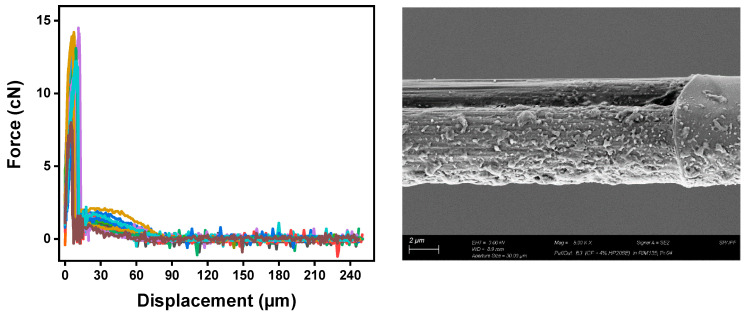
Representative sample (B3) of HP2-06-sized carbon fibers; (**left**) force-displacement curves and SEM observation of a fiber surface after testing showing uniform residual epoxy on the surface (**right**). The colors highlights a set of 20 broken fibers.

The interfacial shear strength (IFSS) of the CF/epoxy resin composites with and without sizing were tested using the single-fiber pullout testing (SFPO) instrument (refer to Section 3.4 for more details). Comparing the force vs. displacement curves of the SFPO tests, carbon fiber sizing levels’ effect on the fiber’s interfacial shear strength is shown in Table 6 and Figure 12, Figure 13 and Figure 14 for an unsized representative sample (A5) of HP3-02 sized carbon fibers and a representative sample (B3) of HP2-06 sized carbon fibers, respectively. Table 6 presents the IFSS of the untreated sample A5 and sample B3 composites, which are 66 MPa, 67.3 MPa, and 68 MPa, respectively. In general, increasing the sizing level increased IFSS. However, it should be noted that even unsized fibers revealed an excellent interaction between the fiber and the epoxy matrix. This finding may be because the epoxy matrix near the fiber is considerably stretched during the pull-out test. On the other hand, the sudden drop in the IFSS at 1 wt.% is due to human-induced damage during the fiber separation process and before the fiber-embedding step.

**Figure 15:**
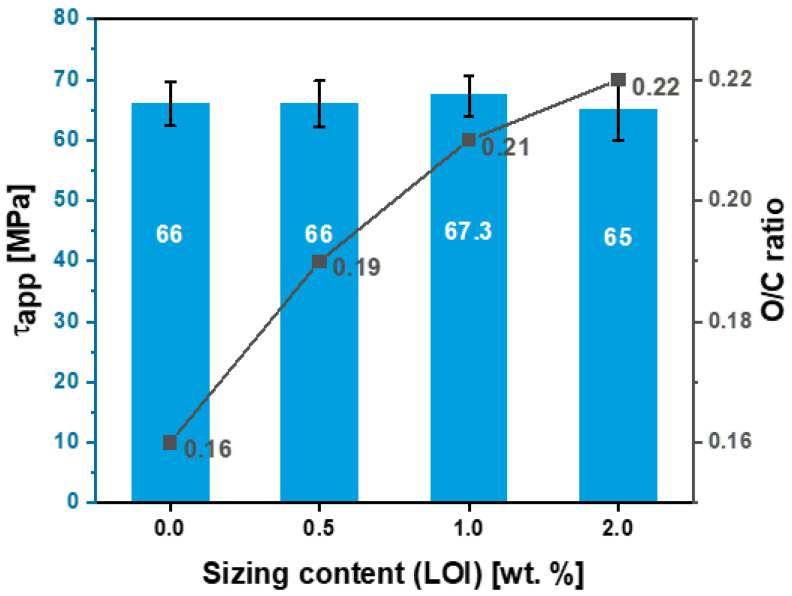
Effect of HP3-02 sizing levels on the apparent interfacial shear stress and O/C surface composition ratio.

**Figure 16:**
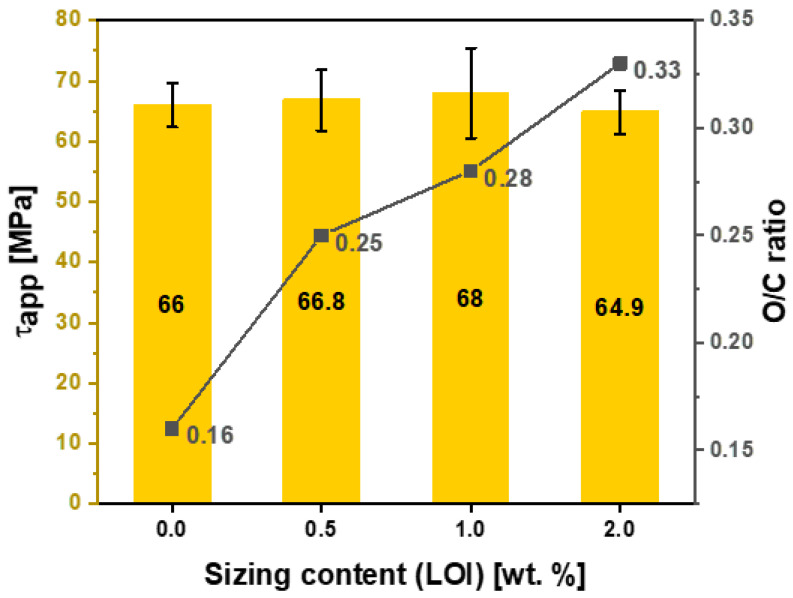
Effect of HP2-06 sizing levels on the apparent interfacial shear stress and O/C surface composition ratio.

The key finding was that despite having the lowest sizing solid percentage (26.3 ± 0.2%), HP2-06-sized carbon fibers had the highest rise in IFSS. A further investigation of fractured fibers by SEM shown in Figure 12, Figure 13 and Figure 14 (right) revealed that HP2-06 sizing resulted in a uniform residual epoxy on the fiber’s surface due to adhesion. Based on XPS analysis, the observed increase in adhesion can be attributed to the growth of hydrophilic oxygenated functional groups on the fiber surface, which are essential to improving their surface adhesion. Figure 15 and Figure 16 confirm the latter claim, where the O/C ratio is increased with an increase in IFSS.

**Figure 17:**
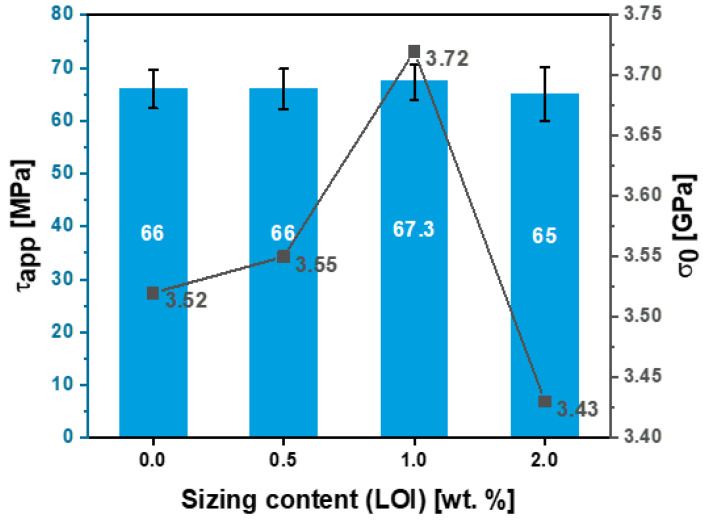
Effect of Hydrosize^®^ HP3-02 sizing content on the apparent interfacial shear stress and characteristic stress of sized and unsized fibers.

**Figure 18:**
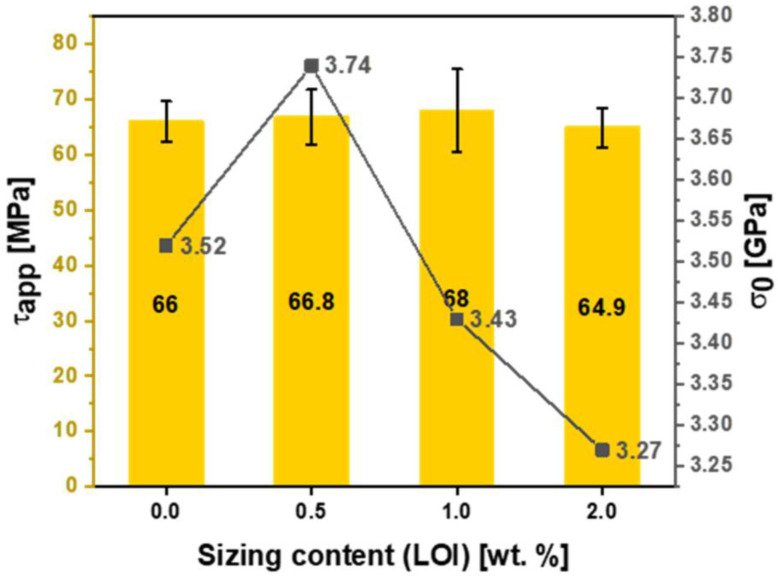
Effect of Hydrosize^®^ HP2-06 sizing content on the apparent interfacial shear stress and characteristic stress of sized and unsized fibers.

For HP3-02 and HP2-06, Figure 17 and Figure 18 demonstrate the application-driven characteristics (tensile and IFSS) at the fiber composite level as a function of the sizing content from a process-by–design space perspective. The desired sizing level content will vary based on the intended end application. For example, for HP2-06 sizing (refer to Figure 18), if the intended use is a shear-driven loading application, then a sizing content of 0.5 wt.% is the optimum sizing level, providing the highest IFSS at 3.74 MPa. On the other hand, if the intended use is a tensile-driven loading application, then a sizing level of 1 wt.% provides the highest tensile stress properties at 68 MPa. These design spaces’ rationalization is important for different reasons: Firstly, it unlocks and utilizes the full potential of the sizing formulations; secondly, different sizing levels provide application-driven cost-saving opportunities. Lastly, it could develop an elegant analytical protocol that can be used to readily characterize the strength and interfacial shear strength of any thermoset or thermoplastic with different sizing formulations and solid content.

## 5. Conclusions

We investigated the effect of fiber sizing levels on carbon fibers’ mechanical properties using a single-fiber tensile test, single-fiber pullout test, and interfacial elemental analysis using X-ray photoelectron spectroscopy (XPS). Two commercially available sizing formulations were applied to the fiber surface. A closed-form solution was developed to calculate the required sizing of solid content to achieve 0.5, 1, and 2 wt.% sizing on the fiber surface. For both sizing formulations, the ultimate tensile strength of the sized carbon fibers showed a 6% increase after sizing. This finding can be attributed to covering the fiber’s surface defects with uniform load transfer along the fiber axis. The XPS result yielded a significant increase in oxygen-containing surface functional groups as the sizing levels increased. We investigated the effect of increasing the O/C ratio on the interfacial shear stress of CF/epoxy composites by conducting 20 SFPO tests. Overall, increasing the O/C ratio resulted in a 6.3% increase in the fiber’s interfacial properties. As a result, two-process by-design spaces were developed to optimize the fiber’s performance. Lastly, generalized fiber sizing and characterization methods were established. The developed methods, equations, and procedures can characterize the strength and interfacial shear strength of manmade fibers with different sizing formulations and solid contents, for both thermoset and thermoplastic matrices.

## Figures and Tables

**Table 1:** Mechanical properties of resin RIMR 135 with curing agent RIMH 137.

Mechanical Data	Value
Density [g/cm^3^]	1.15
Tensile strength [MPa]	70
Tensile modulus [GPa]	2.95

**Table 2:** Breakdown of the sizing solution preparation Using Equation (1).

Sample Name	Sizing Formulation	Solid Content of Film Former (wt.%)	Targeted Lot Mass (g)	DI-Water Diluting Mass (g)	Sizing Level (LOI) (wt.%)
A1	Hydrosize^®^ HP3-02	2	11.73	188.27	0.80 ± 0.06
A2		3	17.60	182.40	1.17 ± 0.03
A3		4	23.47	176.53	1.61 ± 0.05
B1	Hydrosize^®^ HP2-06	2	15.31	184.69	0.46 ± 0.05
B2		3	22.96	177.04	0.81 ± 0.02
B3		4	30.62	169.38	1.02 ± 0.02

**Table 3:** Updated fiber sizing content based on developed models using Equations (4) and (5) and Figure 5 and Figure 6.

Sample Name	Sizing Formulation	Solid Content of Film Former (wt.%)	Targeted Lot Mass (g)	DI-Water Diluting Mass (g)	Sizing Level (LOI) (wt.%)
A4	Hydrosize^®^ HP3-02	1.29	7.57	192.43	0.5 ± 0.03
A5		2.52	14.78	185.22	1.04 ± 0.03
A6		5.0	29.28	170.72	2 ± 0.16
B8	Hydrosize^®^ HP2-06	6.90	52.81	147.19	2.09 ± 0.15

**Table 4:** Values of the two-parameter Weibull distribution function for unsized, 0.50, 1, and 2 wt.% sizing using Hydrosize^®^ HP3-02 and Hydrosize^®^ HP2-06.

Sample Name	Sizing Formulation	Sizing Level [wt.%]	$\sigma_{0}\;[\mathrm{GPa}]$	m	$R^{2}$	n
Unsized fibers (Reference)	NA	0	3.52	6.42	0.98	50
A4	Hydrosize^®^ HP3-02	0.5	3.55	8.48	0.95	50
A5		1	3.72	7.58	0.97	50
A6		2	3.43	5.34	0.98	50
B1	Hydrosize^®^ HP2-06	0.5	3.74	7.51	0.95	50
B3		1	3.27	9.21	0.91	50
B8		2	3.43	7.49	0.98	50

**Table 5:** XPS elemental compositions of unsized and sized carbon fiber surfaces.

Sample Name	Sizing Content (wt.%)	C 1s		O 1s		N 1s		O/C
		B.E./eV	A.C./%	B.E./eV	A.C./%	B.E./eV	A.C./%	
Unsized fiber (Reference)	0	284.80	83.1	532.0	13.2	400.3	2.3	0.16
A4	0.5	284.80	81.4	532.8	15.4	400.1	2.7	0.19
A5	1	284.80	79.5	532.8	17.0	400.0	2.0	0.21
A6	2	284.80	80.5	533.1	17.9	400.4	1.6	0.22
B1	0.5	284.80	78.3	532.8	19.6	400.4	2.1	0.25
B3	1	284.80	77.0	532.8	21.4	400.2	1.5	0.28
B4	2	284.77	74.1	532.5	24.5	399.6	1	0.33

**Table 6:** Interfacial parameters and standard deviations received by SFPO.

Sample Name	Sizing Formulation	Sizing Level [wt.%]	Broken Fibers	$\tau_{app}$ (IFSS) [MPa]	*l_e_* [µm]
Unsized fibers (Reference)	NA	0	0/21	66.0 ± 3.7	79 ± 15
A4	Hydrosize^®^ HP3-02	0.5	0/20	66.0 ± 3.8	80 ± 11
A5		1	0/20	67.3 ± 3.4	75 ± 14
A6		2	0/20	65.0 ± 5.1	80 ± 11
B1	Hydrosize^®^ HP2-06	0.5	0/20	66.8 ± 5.0	73 ± 13
B3		1	0/20	68.0 ± 7.5	78 ± 10
B8		2	0/20	64.9 ± 3.6	76 ± 9

## Data Availability

Data will be made available upon contacting the corresponding author.
